# Who Rules the Cell? An Epi-Tale of Histone, DNA, RNA, and the Metabolic Deep State

**DOI:** 10.3389/fpls.2020.00181

**Published:** 2020-03-05

**Authors:** Jeffrey Leung, Valérie Gaudin

**Affiliations:** ^1^Institut Jean-Pierre Bourgin, ERL3559 CNRS, INRAE, Versailles, France; ^2^Institut Jean-Pierre Bourgin, UMR1318 INRAE-AgroParisTech, Université Paris-Saclay, Versailles, France

**Keywords:** metabolites, epigenetics, acetylation, methylation, histone, acetyl-coenzyme A, S-adenosylmethionine

## Abstract

Epigenetics refers to the mode of inheritance independent of mutational changes in the DNA. Early evidence has revealed methylation, acetylation, and phosphorylation of histones, as well as methylation of DNA as part of the underlying mechanisms. The recent awareness that many human diseases have in fact an epigenetic basis, due to unbalanced diets, has led to a resurgence of interest in how epigenetics might be connected with, or even controlled by, metabolism. The Next-Generation genomic technologies have now unleashed torrents of results exposing a wondrous array of metabolites that are covalently attached to selective sites on histones, DNA and RNA. Metabolites are often cofactors or targets of chromatin-modifying enzymes. Many metabolites themselves can be acetylated or methylated. This indicates that the acetylome and methylome can actually be deep and pervasive networks to ensure the nuclear activities are coordinated with the metabolic status of the cell. The discovery of novel histone marks also raises the question on the types of pathways by which their corresponding metabolites are replenished, how they are corralled to the specific histone residues and how they are recognized. Further, atypical cytosines and uracil have also been found in eukaryotic genomes. Although these new and extensive connections between metabolism and epigenetics have been established mostly in animal models, parallels must exist in plants, inasmuch as many of the basic components of chromatin and its modifying enzymes are conserved. Plants are chemical factories constantly responding to stress. Plants, therefore, should lend themselves readily for identifying new endogenous metabolites that are also modulators of nuclear activities in adapting to stress.

## Introduction

One of the earliest observations redolent of epigenetics was made around 1915 when W. Bateson and C. Pellew made crosses between three “rogue” varieties of pea with its wild-type counterpart. The “rogues” bred offspring of exclusively “rogues”, while the expected wild-type segregants vanished forever (it should be understood that “rogue” at the time was not referring to a specific phenotype, but any off-type with respect to the parent, equivalent to our modern usage of “mutant”). Unusual heritable traits were again noted in the 1950s, more frequently and in different organisms, owing probably to the flourishing discipline of Mendelian genetics. These exceptional cases reported silenced alleles that can go on silencing another allele in *trans*. The causes were variously described as “paramutations” or “transvection effects” (with the benefit of hindsight, each of them represents likely one specific aspect of epigenetics involving *trans*-acting sRNAs). At about that time, the influential embryologist, C. Waddington, also remarked that many developmental traits were not fixed, but subjected to alteration by the environmental conditions (developmental pluripotency). One example was the vein patterns of the *Drosophila* wing, which can be altered by high temperatures during development, yielding a particular *crossveinless* phenotype. If these flies were then maintained for several generations at high temperatures, their progeny became all stably *crossveinless*, even when subsequent generations were returned to normal temperatures. Waddington articulated his ideas on developmental plasticity by using the metaphor of a rugged “epigenetic landscape”. He envisioned that each successive stage of developmental “decisions”, likened to a ball rolling down the landscape, can be nudged by environmental cues to deviate from its original path down a different furl (pathway). The landscape itself was also not static, but can change shape, as its floppy crust (can be equated with chromatin) is suspended over numerous genes whose actions are similar to those of pulleys, tugging asynchronously at the landscape. The term “epigenetics” was coined by Waddington as a nod to the existence of phenotypic determinants above the well-accepted Mendelian entity later called genes (Slack, 2002; Stam and Mittelsten Scheid, 2005; Noble, 2015).

These ideas did not gain immediate traction, as they of course conjured up Lamarckian inheritance, a forbidden fruit in the Mendelian Eden. Ironically, some of the molecular components for his model were already being discovered by his contemporaries at Cambridge University, his alma mater where he taught embryology. Between the 1950 and 1970s, Cambridge was the scientific mecca for some of the most brash and brilliant minds that ever united under the same proverbial roof, trying to understand life’s workings by blurring the intellectual divide between biology, chemistry and physics (https://qesp.org/james-watson-francis-crick-maurice-wilkins-and-rosalind-franklin/). This new reductionist approach, called molecular biology, was inaugurated there by a series of ground-breaking discoveries (e.g. the double-helix) and equally reverberating technical achievements–the most notable being DNA sequencing–that laid down the path for today’s genomics. In fact, the Watson–Crick DNA structure, with its full mechanistic implications for Mendelian inheritance, was published 4 years ahead of Waddington’s matured ideas in his book The Strategy of the Gene [reviewed by (Slack, 2002)]! Following the wake of the double-helix, acetylation on histones was also turning up a mere 100 km from Cambridge. These fractions of histone hydrolysates might have been annoying to work with, as they would thwart routine precipitations, owing to their increased hydrophobicity (Phillips, 1963). With the inexorable progress of molecular biology–debuting in the late 70s rsquo;s and its eclosion into the now cutting-edge genomics–epigenetics and the other lesser-used sobriquets like paramutations are understood to involve the same phenomenon of phenotypic changes without DNA mutations. These (epi-)phenotypes are caused by rearranged chromatin landscape triggered by chemical attachments to the DNA and histones, or in some gene-silencing systems, through the intermediate steps of *trans*-acting small RNAs that recruit the DNA- and histone-modifying enzymes. The reconfigured chromatin landscape, in turn, leads to altered accessibility to the genes underneath by the transcriptional machinery.

**Figure 1:**
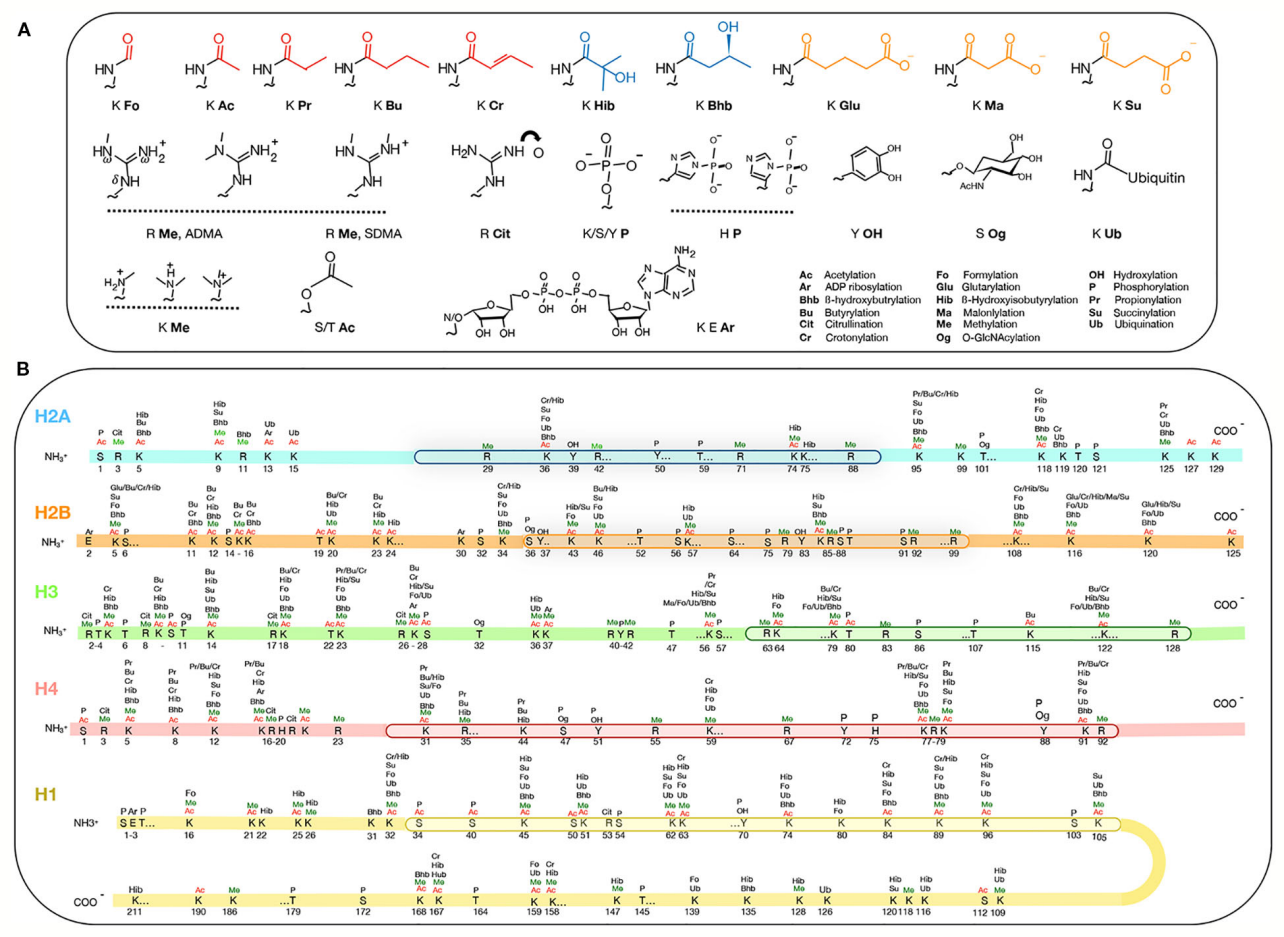
Post-translational modification of histones. **(A)** Metabolites attached to histones. The top row shows acyl groups that are hydrophobic (red), polar (blue) and charged (orange) attachments. **(B)** The types and amino acid positions of the modifications on the core and the linker H1 histones. The globular domains (by which the histones themselves interact) are boxed. Figure is modified from Huang et al., 2014; Sabari et al., 2017.

A current resurgence of excitement in epigenetics surrounds the question of how it can be extensively controlled by metabolism. This seemingly esoteric question is in fact revived by the awareness that unbalanced diets can cause many diseases due to deregulated epigenetic mechanisms, leading then, of course, to misexpression of genes (Kinnaird et al., 2016). Some cancers have been traced to malfunctioning RNA-modifying enzymes and the enthusiasm to understand the RNA code was buoyed by the hopes of developing treatments targeting the modifying enzymes. Along the way, the field of “epitranscriptomics” dedicated to understanding RNA modifications was launched (Saletore et al., 2012). Many of the metabolites are also donors, inducers, inhibitors, substrates, and co-factors of chromatin remodeling enzymes. There are over 260 sites on the animal histones (H1, H2A, H2B, H3, and H4) that are known to be modified post-translationally [**Figure 1**; see (Huang et al., 2014; Sabari et al., 2017)] and probably nearly so for plant histones. Benefitting from the Next-Generation technologies, the number of potential sites and the type of chemical adducts are rapidly expanding. One generality is that the vast majority of the histone modifications belongs to the class called short-chain acylation, defined by a thioester bond [R-C(=O)-S-R’] that links one molecule (e.g. lysine in a histone) to the next (e.g. acyl adducts) (**Figure 1**). For example, the widely-studied acetyl adduct is linked to the ε-amine of the lysine (K) by acylation. The other less-known acyl groups are distinctive by their hydrocarbon chain length, hydrophobicity or net electrical charges.

In plants, studies directly addressing the functional relationships between metabolism and epigenetics are still relatively rare (Shen et al., 2015; Shen et al., 2016). But because many metabolites, histones, and modifying enzymes are highly conserved, the general principles derived from studies of other models should nevertheless provide useful clues into plants. On the other hand, because the lifestyle of plants is different from these other organisms, experimental confirmation or refutation is still ultimately desirable, if not necessary. In particular, plants produce myriads of secondary metabolites, many of which are implicated in defense against pathogens and abiotic stresses, as well as being immensely important for human health. Plants should thus be an excellent model to address the question of how these metabolites can relay environmental changes to the nucleus, a timely topic in view of global warming. This review will articulate mainly around acetylation and methylation, as they provide most of the current knowledge. This owes to the fact that most of their relevant modifying enzymes are known. It generally holds that the overall cellular concentrations of acetyl-coenzyme A (acetyl-CoA) and S-adenosylmethionine are the rate-limiting suppliers of, respectively, acetyl and methyl groups. So far, the literature available on the potential competitors for these two adducts in both plant and animal models is frequently limited to DNA and proteins (e.g. chromatin and the overall proteome). Plant epigenetics elevated to the scale of epigenomics will need to step outside the provinces of just protein and DNA. The complete acetylome and methylome in a cell must include all molecules requiring these same adducts for functioning. For this reason, we are broadening this discussion here to include epi-transcriptomics and “epi-metabolites”, by drawing in examples from both plant and non-plant models. One thing we try to avoid is lengthy coverage on how histone acetylation, histone methylation, and DNA methylation specifically influence plant development, especially flowering, as these areas have been amply covered. To help orientate non-experts, the following reviews should be highly informative: Zilberman et al. (2007), Law and Jacobsen (2010), Liu et al. (2010), Matzke and Mosher (2014), Liu et al. (2016), Trindade et al. (2017), Wang and Kohler (2017) and Friedrich et al. (2019).

### In Plants, are the Cellular Concentrations of Acetyl-CoA Commensurate With the Level of Histone Acetylation?

**Figure 2:**
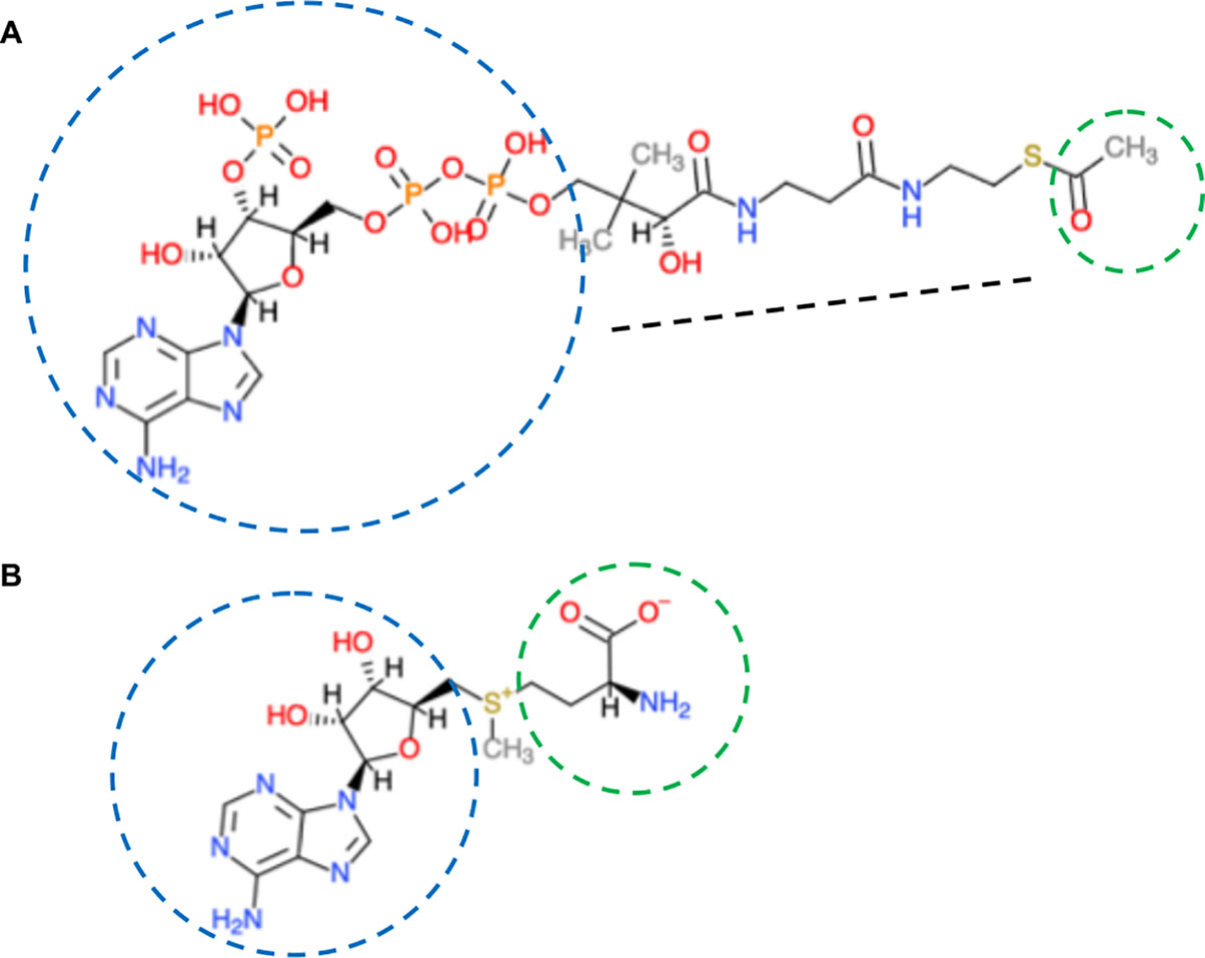
Co-enzyme A and S-adenosylmethionine structures. **(A)** Co-enzyme A. Composed of an aliphatic chain (black dotted line), joined to adenosine diphosphate (blue circle). The sulfhydryl group (–SH) at one end is the most reactive. It can bond with, via the thioester with carboxylic acids (RCOOH), the most important is acetic acid (CH3COOH; acetyl is in green circle). Co-enzyme A has two major functions-as an energy carrier because of the high-energy phosphates and to transport two-carbon units in the form of acetyl between various biological molecules. **(B)** S-adenosylmethionine is synthesized from ATP (adenosyl in blue) and the sulfur-containing amino acid methionine (green circle). The activated methyl group (red) is linked to the sulfur (yellow). Chemical molecules were derived from ChemDoodle Web Component 2D Sketcher.

Acetyl is used by cells as the basic currency for circulating two-carbon units in metabolic cycles. In eukaryotes, it comes exclusively from acetyl-CoA (**Figure 2A**) and several plant homologs of acetyl-CoA biosynthetic genes have been reported: pyruvate decarboxylase, acetaldehyde dehydrogenase, acetyl-CoA synthase, plastid pyruvate dehydrogenase, and ATP-citrate lyase. (https://portal.nifa.usda.gov/web/crisprojectpages/0187429-how-is-acetyl-coa-generated-in-plants.html). Many of these have not been extensively studied.

**Figure 3:**
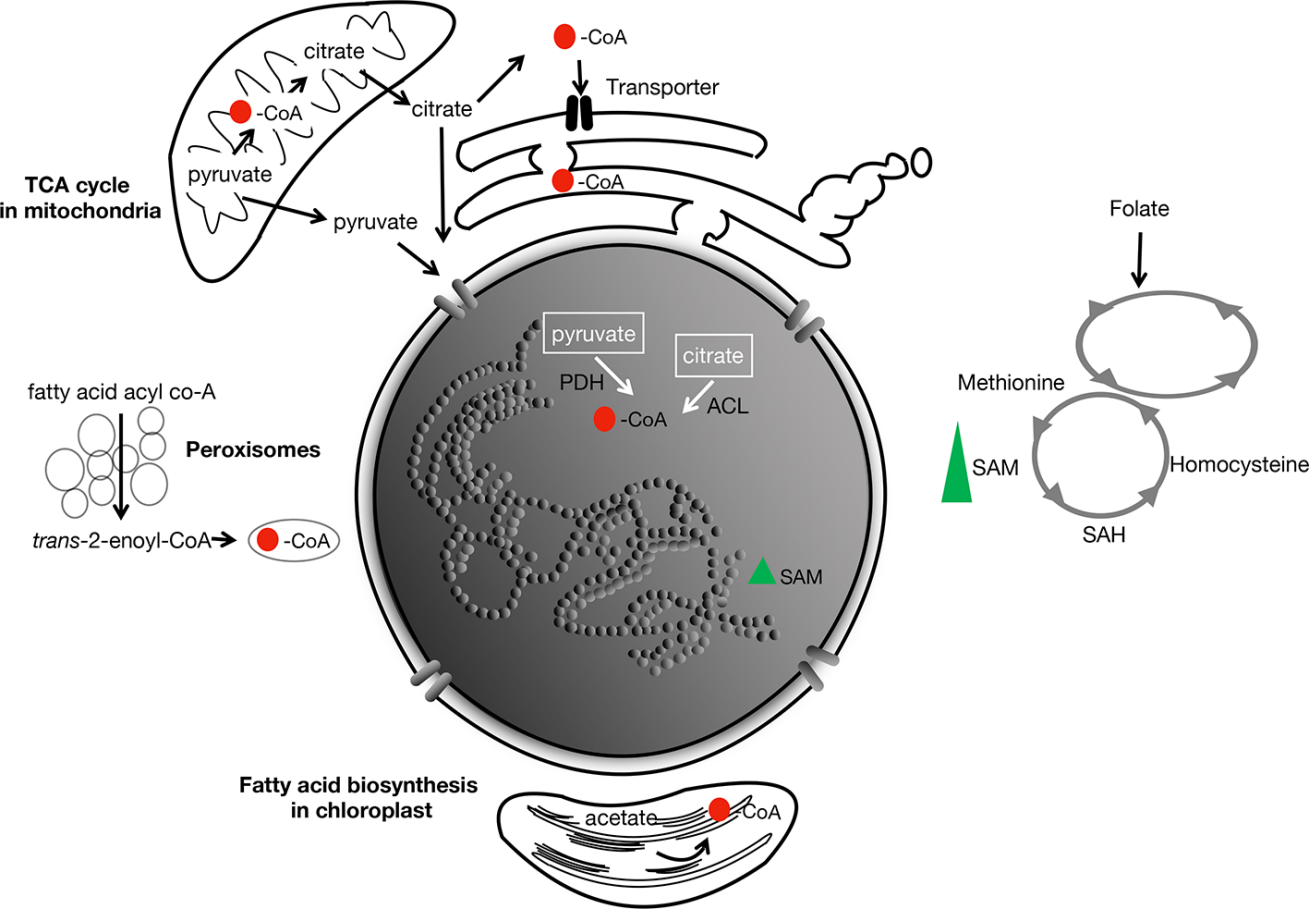
Main metabolites involved in acetylation and methylation in the cell. CoA is produced by the TCA cycle in the mitochondria, and during fatty-acid biosynthesis in the chloroplast and peroxisomes. Its redistribution to the different subcellular compartments is hypothesized to be by diffusion (as pyruvate and citrate from the mitochondria) across membranes, or perhaps through nuclear pores. In animals, transporters of acetyl-CoA in the endoplasmic reticulum has been described (Jonas et al., 2010; Hirabayashi et al., 2013). Red spot, acetyl; PDH, pyruvate dehydrogenase; ACL, ATP-citrate lyase. SAM, the rate-limiting methyl group supplier is derived from methionine and the folate cycle. Green triangle, SAM.

In other model organisms (e.g., yeast, mammals), the global level of acetylated histones has been shown to be correlated positively with that of acetyl-CoA in the cell. This suggests that acetyl-CoA availability likely represents the bottleneck. Acetyl-CoA itself is not membrane permeable so that within a compartmentalized cell, there are likely local heterogeneities in concentrations. Its flux among the subcellular compartments is accomplished rather by the membrane-permeable citrate and pyruvate, generated from the TCA cycle in the mitochondria (**Figure 3**). It has been hypothesized that membrane compartmentalization could in fact contribute to target specificity of some acetyltransferases (van Roermund et al., 1995; Jonas et al., 2010; Kistler and Broz, 2015; Sivanand et al., 2018; Wang et al., 2019).

Chen et al. (2017) have identified mutations in the Arabidopsis *ACETYL-COA CARBOXYLASE 1* (*ACC1*) locus, whose encoded enzyme converts cytosolic acetyl-CoA into malonyl-CoA required for the elongation of fatty acids produced in the plastids. While *acc1* null mutants are embryo lethal, leaky mutants show variable symptoms of vegetative growth defects in addition to a reduced stature, coined collectively as “bonsai phenotypes” (Fatland et al., 2005). The leaky *ACC1–5* contained about 50% higher amount of acetyl-CoA, and this is correlated with a global 50%–70% increase in histone acetylation over hundreds of chromosomal sites.

This semi-randomness in acetyl distribution over the chromosomes is somewhat expected, but at the level of individual histones, the lysine acetylation pattern showed a surprisingly strong bias. Instead of many lysines being eligible recipients owing to the higher acetyl-CoA import into the nucleus, the only notable beneficiary was H3K27. Seven other lysines (H3K9/14/18 and H4K5/8/12/16), known sites of acetylation, had levels indistinguishable from that of the wild type. Although there are many other possible target lysines or even arginines that have not been examined, this small size sample is nonetheless sufficient to hint at a rather strong selectivity in H3K27 to be acetylated. The underlying reason for this bias is not known.

The cytosolic acetyl-CoA biosynthetic enzyme ATP-citrate lyase (ACL) mentioned above is composed of two distinct subunits, ACLA and ACLB. The holoenzyme is a hetero-octomer composed of four subunits each of the A and B. Genetic analysis on *acl* mutants, because of their severe phenotypes or even lethality, indicated that the ACL complex represents a rate-limiting step or “non-redundant” generator of cytosolic acetyl-CoA (Fatland et al., 2005). This interpretation is consistent with the observation that hyperacetylation at H3K27 in *ACC1* can be partially blocked by expressing a ß-estradiol inducible artificial microRNA targeted against *ACLA*. Thus, it is clear that acetyl-CoA homeostasis and the global histone acetylation levels are intrinsically linked. This further implies that acetyl-CoA in the cytosol can cross the nuclear membrane, possibly by passive diffusion via the pore complexes (Shen et al., 2015).

Genetic and biochemical analyses on eight of the 12 acetyltransferases in Arabidopsis revealed that the hyperacetylation of H3K27 in *ACC1* depended on *At3g54610*, the single-copy gene encoding the nuclear histone acetyltransferase (HAT) GCN5/HAG1 [hereafter GCN5; for classification and descriptions of HATs, see (Shen et al., 2015)]. The argument that GCN5 is the major writer of H3K27ac is reinforced by the observations that the (hyper)acetylation at H3K27 in *aac1-5* was abolished in the double mutant *gcn5 aac1–5*. This was also accompanied by partial reversion of one of the phenotypes, “leaf fusion”, observed in the single mutant *aac1–5* (Chen et al., 2017).

### How Does Acetyl-CoA Metabolism Link Up to Chromatin? Early Clues From Genetic Analyses

In Arabidopsis, genetic screens for suppressors of gene silencing based on altered expression of reporter transgenes *ProRD29:LUC* and *Pro35S:NPTII* (confers the kanamycin resistance) had identified loci in metabolism. One suppressor mutant showed kanamycin-sensitivity (due to hypermethylation of the *NPTII* gene), but still expressing *LUC*. The suppressor mutation was mapped to *At3g51840*, whose gene sequence predicted a peroxisomal acyl-CoA oxidase 4 (ACX4) involved in fatty acid ß-oxidation (Wang et al., 2019). Acyl-CoA oxidases in general act on CoA derivatives of fatty acids. In the case of ACX4, it catalyzes the conversion of fatty acid acyl-CoA [chemical formula: –C(O)–fatty acid] to *trans*-2-enoyl CoA, which is eventually converted into acetyl-CoA [chemical formula: –C–(O)–CH_3_] (**Figures 2A** and **3**). This is thought to be the predominant way by which the rate of acetyl-CoA flux is controlled through ß-oxidation in peroxisomes. The acetyl-CoA level was lower in this mutant than in the wild type. By specific antibody staining in nuclei cytological spreads, histones H2B, H3, and H4 were all found to have lower than wild-type levels of acetylation levels. When histone H3 was analyzed in detail, again, some bias in the target lysines was detected. It was found that H3K9ac, H3K18ac, and H3K23ac were moderately reduced, those of H3K14Ac and H3K27Ac were unchanged. Over-expression of the above-mentioned *ACLA* and *ACLB* simultaneously (to reconstitute the complete cytosolic biosynthetic complex), but not the subunits individually, led to the rescue of the kanamycin-sensitivity of *acx4*. These results uncovered that ß-oxidation of fatty acids in peroxisome is interlocked with the regulation of histone acetylation and DNA methylation relevant to gene silencing.

The control of circadian rhythm in animals depends on one of the central regulators of circadian rhythm, CLOCK (Circadian Locomotor Output Cycle Kaput), which unexpectedly turns out to be a specific histone acetyltransferase (Doi et al., 2006; Eckel-Mahan and Sassone-Corsi, 2013). We mentioned this here because it was largely this finding that consolidated the idea that the epigenetic regulatory mechanisms are fundamentally controlled by metabolism. Counterbalancing CLOCK’s activity are the repressors, PER and CRY. These can form heterodimers in the cytosol, then migrate into the nucleus where they physically block the CLOCK complex from binding to target chromatin sites. PER2:CRY1 dimerization is sensitive to the level of polyamines. And in the mouse, the decline in polyamines is correlated with the lengthening of the circadian period (Zwighaft et al., 2015). Polyamines also stimulate histone acetylation in several mammalian cell types, although the mechanistic contour remains hazy (Hobbs and Gilmour, 2000).

### Metabolic Status of the Cell and Histone Deacetylases

The cofactor nicotinamide adenosine dinucleotide [NAD(+)] was initially discovered as an electron carrier (becomes NADH) in the oxidation of carbohydrates. The involvement of NAD(+) in histone modification is just beginning to be explored in plants. In combination with the *de novo* synthetic pathway (from aspartic acid), Arabidopsis has a salvage pathway in which nicotinate mononucleotide is regenerated from nicotinamide. In animals, the Sirtuin-Like proteins SIRT 1 and SIRT6 form a class of histone deacetylases whose activities depend on NAD(+) (so-called class III HDACs). Sirtuins are activated at times of energy deficit and reduced carbohydrate energy, associating with high NAD(+) levels. The activities of SIRT seem to be, moreover, limited by NAD(+) availability (Pacholec et al., 2010; Gerhart-Hines et al., 2011; Hirschey et al., 2011; Fischer et al., 2012). Like acetyl-CoA, the levels of NAD(+) could, therefore, act as a signal that communicates the cellular redox status to the chromatin, through activating specific SIRT’s (in mammals, these are SIRT1, SIRT6, and SIRT7).

Mammalian HDACs are inhibited by ß-hydroxybutyrate, a ketone that has been known primarily as a circulating source of energy in response to fasting. ß-hydroxybutyrate can thus logically act as a trigger inside cells to increase the global levels of histone acetylation and the expression of numerous genes, particularly those encoding oxidative stress-protection factors (Shimazu et al., 2013). Whether ß-hydroxybutyrate exists in this form freely in plants is not conclusive, but poly-ß-hydroxybutyrate is present (Tsuda et al., 2016). Whether this can metabolize to ß-hydroxybutyrate is not known.

### Polyamines and Histones Cooperate in Remodeling the Chromatin Structure

Polyamines are aliphatic compounds of low molecular weight bearing more than one amino group. Because of the amino groups, at physiological pH, these compounds are therefore polycations. The four most abundant, or universal, polyamines are putrescine, spermine, spermidine, and cadaverine, which are found in disparate ratios in virtually all cellular life forms, ranging from prokaryotes (e.g. Mycoplasma, *E. coli*, Salmonella, etc.) to humans.

In eukaryotes, one of the earliest roles attributed to polyamines is that they interact with the chromatin (Morgan et al., 1987). Polyamines are ancient molecules that played a role in packaging DNA and RNA in simple cells and viruses. Although most of the demonstrations on polyamine–DNA interactions have been done *in vitro*, there are genetic evidences, especially obtained from yeast, entirely consistent with these interpretations.

The mutation *gcn5* disrupts the yeast homologous histone acetyltransferase and impairs the transcriptional activation of many target genes (Xue-Franzen et al., 2010). The mutant is also hypersensitive to oxidative stress and shows retarded growth (Pollard et al., 1999). In genetic screens for extragenic suppressor mutations, the *gcn5* phenotypes were found to be partially restored to those of the wild type by the overexpression of *ARG3. ARG3* does not encode chromatin-related proteins, but ornithine decarbamylase, which converts the non-protein amino acid ornithine to citrulline, an intermediate in the urea cycle as well as an arginine derivative found in histones (**Figure 1**). Because ornithine is the limiting step in polyamine biosynthesis, its catabolic conversion into citrulline competes with its alternative production of polyamine. This led to the hypothesis that polyamine deficiency caused by *ARG3* overexpression should suppress the histone acetyltransferase deficiency. This was indeed confirmed. Similarly, another mutation, the loss-of-function *spe1* can also partially reverse the effect of *gcn5* based on reporter-gene expression assays (e.g. *HO-lacZ*, *SUC2-lacZ*, etc.). *SPE1* encodes ornithine decarboxylase, which converts ornithine into putrescine, a precursor for the biosynthesis of other polyamines. Finally, combining *spe1* and a semi-dominant allele of histone H4 (*hhf2-7*; which impairs nucleosome-mediated gene repression) almost completely alleviated GCN5-dependent expression of the reporter gene. These results, together, provide strong genetic proofs that the normal roles of polyamines include the repression of gene expression through interactions with nucleosomes. *In vitro*, spermidine can efficiently promote oligomerization of nucleosomes onto DNA. Core histones acetylation attenuates this process (Pollard et al., 1999).

Both core histones and polyamines seem to be common targets of transglutaminases (Folk et al., 1980; Ballestar et al., 1996). These enzymes catalyze the additions of amine groups to the amino acid glutamine. Histones are easily cross-linked or modified by polyamines *in vitro* and raises the possibility that they are transglutaminase substrates *in vivo* (Nunomura et al., 2003). Human histone proteins directly purified from HeLa cells do show abundantly linked putrescine, spermidine or spermine (Yu et al., 2015). More unexpected is that serotonin, a neural transmitter as well as a trophic factor that helps neurons to grow, is added by a specific transglutaminase onto glutamine 5 of H3 (Farrelly et al., 2019). And this modification can take place only if H3K4 is already trimethylated, attesting to the functional specificity of this serotonin mark. It is also worthwhile to emphasize that this discovery portents that other neural transmitters, such as dopamine and histamine, could well be histone marks (Cervantes and Sassone-Corsi, 2019). Serotonin (Erland and Saxena, 2017), dopamine (Bell and Janzen, 1971) and histamine (Sanchez-Perez et al., 2018) (https://www.britannica.com/science/histamine) all exist in plants, portending the astonishing diversity of adducts that are still waiting to be uncovered.

### Mechanisms of Acetylation of Metabolites and Histones Hint at Early Co-Evolution

Genomic-scale analysis of “acetylome” has found that many non-histone proteins, including those in organelles, are acetylated. Some of the acetylated non-histone proteins have been described in Shen et al. (2015), as potential stakeholders of the same acetyl-CoA source.

Beyond proteins, the more intriguing point is that many metabolites themselves are targets of acetylation, prompting some to call these epi-metabolites (Showalter et al., 2017). For examples, some amino acids, neurotransmitters or polysaccharides such as glucosamine and muramic acid are all substrates of acetyl-CoA-dependent acetyltransferases (Engelke et al., 2004; Vetting et al., 2005; Han et al., 2012; Showalter et al., 2017; Christensen et al., 2019).

Acetyltransferase activities have been reported to co-purify from mammalian cell extracts that can acetylate *in vitro* both histones and polyamines, although the nature of these proteins is not known (Wong et al., 1991; Matthews, 1993). Equally intriguing is whether some acetyltransferases can modify both histones and metabolites, with implications for the evolution of this class of enzymes. One recent discovery is that the activity of the human H3 acetyltransferase, PCAF, can be enhanced towards histone H3 (10 µM) by the presence of low concentrations (<5 µM) of *N*^8^-monoacetylspermidine i*n vitro*. It was hypothesized that the PCAF Bromodomain is able to bind *N*^8^-monoacetylspermidine, which then increases the affinity of the acetyltransferase towards H3. In the presence of high spermidine concentrations (>15 µM), PCAF switches substrate preference, exhibiting *N*^8^-spermidine acetyltransferase activity over that of histone, resulting in *N*^8^-monoacetylspermidine (Burgio et al., 2016).

So far, there is no polyamine deacetylase known from eukaryotes. However, acetylpolyamine amidohydrolases (APAH) with deacetylase activity had been isolated from the soil bacterium *Mycoplana ramosa* (formerly *bullata*) (Fujishiro et al., 1988; Sakurada et al., 1996). These enzymes, dimeric, have a strong affinity towards acetylated spermine, *N*^1^– and *N*^8^ – isoforms of spermidine, cadaverine, and putrescine. The APAH has been crystallized and its structure resembles that of a HDAC–like oligomer. In fact, APAH displayed significant activity towards L–Lys (ε–acetyl)–coumarin, the small *in vitro* substrate for HDAC, but reduced activity towards the larger HDAC test substrate acetyl–L–Arg–L–His–L–Lys(ε–acetyl)–L–Lys(ε–acetyl)–coumarin. Dimerization is important, because the interface creates an “L”-shaped active site tunnel would allow only substrates that are sufficiently narrow and flexible to enter. These results also hint at the possibility that a comparable dimeric HDAC could catalyze polyamine deacetylation in eukaryotes (Lombardi et al., 2011). The implication is that prokaryotic polyamine deacetylases might have been the predecessors that eventually evolved into eukaryotic histone deacetylases.

### Histone Methylation and Metabolic Cofactors

Methylation of histones and DNA is well known, but their methylation status is coordinated with the metabolism. On a genome-scale, methylome will need to explore beyond histones and DNA, by taking into account of other cellular elements, including RNA and metabolites.

Approximately 1% of the genes in several reference eukaryotic genomes, ranging from Arabidopsis to mammals, encode proteins with motifs characteristics of methyltransferases (Katz et al., 2003). This relatively large number of predicted methyltransferases suggests there might be an equally large trove of corresponding methylated substrates yet to be discovered. By far, the most characterized methyl acceptors are histones (**Figure 1**) and DNA, with direct consequences on the epigenetic regulation of gene expression.

The universal methyl (–CH_3_) donor is S-adenosylmethionine (SAM) (**Figure 2**). SAM originates from folate-dependent one-carbon metabolism (the transfer of one-carbon units) (**Figure 3**). The vitamin B_12_-dependent methionine synthase uses 5-methyl-tetrahydrofolate (5-CH_3_-THF) as the one-carbon donor for the methylation of homocysteine to methionine, the precursor of SAM. Removal of the methyl moiety from SAM results in S-adenosylhomocysteine, which is a competitive inhibitor of methyltransferases. On the other hand, decarboxylation of SAM to S-adenosyl-methioninamine contributes the α-aminopropionyl group to the biosynthesis of spermine and spermidine, as well as being the precursor of certain amino acids and ethylene (Miyazaki and Yang, 1987; Roje, 2006).

Histone methylation is carried out by families of transferases represented by SUPPRESSOR OF VARIEGATION SU(VAR)3-9, ENHANCER OF ZESTE [E(Z)], TRITHORAX (Trx), and ASH1 (absent, small, or homeotic discs 1). These protein families share a common stretch of 130 aa, called the SET domain, an acronym based on the founding members of the first three families, identified in Drosophila by genetic screens for modifiers of variegating phenotypes (see section *HETEROCHROMATIN PROTEIN1 and Histones-Collaborators or Competitors?*). Histone methyltransferases in Arabidopsis can control the expression of genes with functions in metabolism. For example, the SET-domain SDG8 catalyzes H3K36me3 in gene bodies, inducing high-level expression of a specific set of light- and/or carbon-responsive genes important for photosynthesis, metabolism and energy production (Li et al., 2015).

The catalytic activities of histone demethylases are directly dependent on metabolites as co-factors. The first type is the amine oxidases, represented by the Lysine-Specific Demethylase1 (LSD1), whose activities require flavin adenine dinucleotide (FAD). In Arabidopsis, several homologs (e.g. *FLD*, *LDL1*, *LDL2*) play a prominent role in flowering time through the suppression of *FLC* (Jiang D. et al., 2007; Liu et al., 2007).

In mammals, histone demethylases have extremely broad functions, covering many metabolic pathways. For example, high carbohydrates will stimulate FAD accumulation, which can then activate LSD1 and also the deacetylase Sirtuin. Thus, it seems that nutrient signals are transduced by at least two epigenetic pathways, implicating both demethylation and deacetylation. LSD1 is known to demethylate the repressive mark H3K4me2 on the fatty acid synthase gene, to activate its expression. But it can also inhibit glucogenesis by demethylating the active mark H3K4me2 on *FBP1* and *G6Pase* (Nakao et al., 2019). LSD1 and many other epigenetic components are also implicated in DNA metabolism, in which chromatin structures at double-strand breaks are remodeled to allow DNA-repair machinery to access the spatially confined region surrounding the double-strand DNA break [for example, see (Wei et al., 2012)].

The other class of demethylases is a complex family of proteins characterized by the Jumonji C (JmjC) domain, with members targeting specific histone lysines at different methylation states. Their catalytic activities need ferrous iron [Fe(II)] and 2-oxoglutarate (2-OG). Because Fe(II) is sensitive to reactive oxygen species (ROS) produced during aerobic metabolism and oxidative stress, JmjC activities might thus be modulated by Fe(II) availability.

There is also a sizeable number of arginine (R) residues on H3 and H4 that are targets of methylation [which will not be enumerated here; for details, see (Zhang and Reinberg, 2001; Liu et al., 2010; Ahmad and Cao, 2012)] (**Figure 1B**). The essential point here is that this amino acid bears three guanidino nitrogen atoms (CH_5_N_3_)–two terminal nitrogens designated as *ω* and one internal designated as *δ* (**Figure 1A****)**. Contingent on the methylation positions on the nitrogens, one isomer generated is called SDMA, or symmetric dimethylarginine, in which each of the two *ω* guanidino nitrogens is bound to one methyl group; the other is designated ADMA, or *ω*-*N*^G^, *N*^G^ asymmetric dimethylarginine, in which two methyl groups replace the two hydrogens of the same *ω* guanidino nitrogen atom. These dimethylarginine isomers, ADMA and SDMA, seem to generate contrasting biological readouts. In mammals, ADMA at H4R3 is associated with transcriptional activation (Wang et al., 2001), whereas SDMA at the identical position is a repressive mark (Zhao et al., 2009). The regulatory readouts dictated by these modifications do not stop here, but subsequent proteolysis of the methylated proteins liberates ADMA and SDMA. ADMA (but not SDMA) then turns into a metabolite inhibitor of the endothelial nitric oxide synthase (Xuan et al., 2016). Arabidopsis arginine methyltransferase homologs exist (Liu et al., 2010), it is likely that ADMA might also act as a metabolic inhibitor.

### DNA Modifications–Reversible Methylation, Novel Bases, Double-Strand DNA Break, Gene Silencing

Studies on DNA modification have been largely focusing on methylcytosine. It was first reported in 1925 in *Mycobacterium tuberculosis*, but it is now widespread in most model organisms, except *C. elegans*. DNA methylation is found on nucleotides (*N*^6^-)methyladenine, (*N*^4^- and *N*^5^-)methylcytosines (Ratel et al., 2006). In Arabidopsis, *N*^5^-methylcytosines are abundant and present in CG, CHG and CHH sequence contexts. These marks are catalyzed and maintained by a battery of enzymes leading to restructured chromatin (e.g. MET1, DDM1, SWI2/SNF2, CMT2, CMT3, DRM2, etc.). The present-day technologies, however, are not of sufficient resolution to pinpoint the specific function of the individual types of methylated nucleotides. For now, it appears that it is rather the optimal levels of DNA methylation are important for plant growth (Dowen et al., 2012; Zhang et al., 2018; Gonzales and Vera, 2019).

Double-strand DNA breaks (DSB), which is mechanistically associated with DNA repair and recombination, is best correlated with low DNA methylation levels. In meiosis, during which recombination has been explored in detail, it starts with a double strand break (DSB) introduced in the chromosomal DNA by SPO11-1, SPO11-2, and MTOPVIB in a topoisomerase IV-like complex. Sequencing the oligonucleotides recovered from SPO11-1 revealed hotspots that are clearly low in cytosine methylation, gene-rich, and tend to exclude nucleosomes as ascertained by micrococcal nuclease digestion (Choi et al., 2018). Similar analyses had also been carried out in maize, by taking advantage of the RAD51 protein, which binds to sequences near DSBs (He et al., 2017). These RAD51-bound sequences are not skewed towards gene-rich regions but rather dispersed throughout the genome, including centromeric and pericentromeric regions. In fact, 75% of DSBs are derived from repetitive DNAs and retrotransposons. Despite these differences relative to Arabidopsis, maize DSBs are also found in DNA regions of low methylation as well as being nucleosome-free (He et al., 2017).

**Figure 4:**
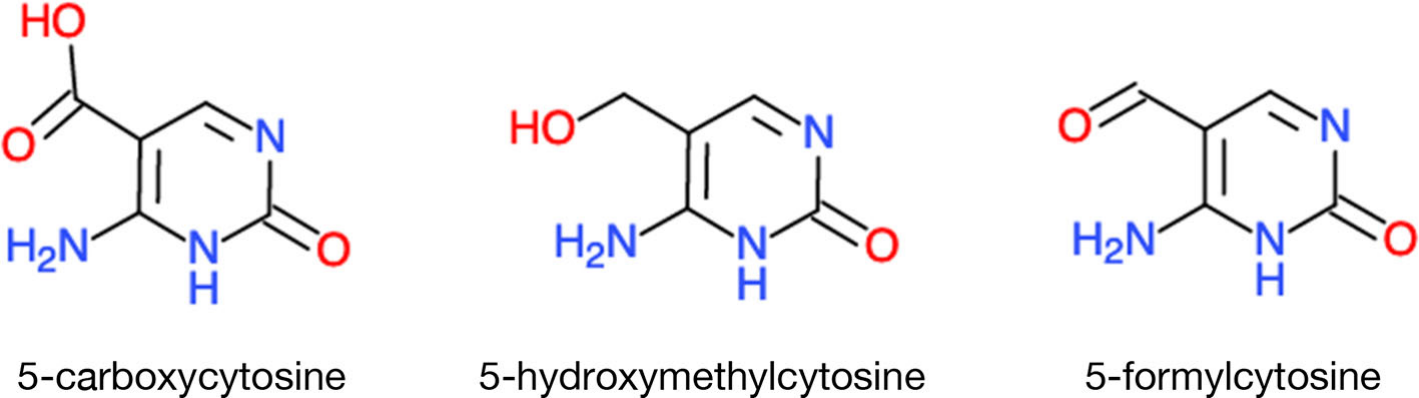
A sample of unusual bases in eukaryotic DNA. TET DNA demethylases, instead of simply removing the methyl groups, they leave in their wake rearranged nucleotides. Chemical molecules were derived from ChemDoodle Web Component 2D Sketcher.

DNA methylation is reversed by demethylases belonging to a family of DNA glycosylases but the relationship with metabolism has not been firmly established, especially in plants. In animals, one enzyme family is called TEN-ELEVEN TRANSLOCATION METHYLCYTOSINE DIOXYGENASEs (TETs). TET converts 5-methylcytosine to 5-hydroxymethylcytosine, 5-formylcytosine, and 5-carboxylcytosine; all of these unusual base derivatives have been found in the mouse genome (Ito et al., 2011) (**Figure 4**). TET activities–like those of histone deacetylases and demethylases–share the cofactors Fe^2+^ and α*-*ketoglutarate. Another link between DNA methylation/demethylation and metabolism is through vitamin C. Its simple addition to the culture medium can induce extensive epigenetic reprogramming, converting mouse fibroblast cells back into pluripotent or stem cells; the efficiency of this conversion is TET1-dependent (Pera, 2013).

The above methyl-cytosine derivatives are not the only novelties found in mammalian genomes. In fact, eukaryotic nuclear DNA could be more extensively modified than we realize (https://en.wikipedia.org/wiki/DNA). For now, most of the modified bases have only been reported in microorganisms, such as bacteria and phages, but some of the “exotic” modifications might still turn up in eukaryotic genomes: Uracil was first found in the genomic DNA of Plasmodium, but it is now present on at least two human chromosomes (Shu et al., 2018) (**Table 1**). Obviously, how all of these unusual bases are maintained and escape correction mechanisms is not known. Neither do we know whether a parallel exists in plants.

**Table 1:** Modifications and substitutions of DNA bases.

Bases	Modifications
Adenosine	*N^6^*-carbamoyl-methyladenine
Guanine	7-methylguanine
Cytosine	*N*^4^-methylcytosine
	5-carboxylcytosine
	5-formylcytosine
	5-glycosylhydroxymethylcytosine
	5-hydroxycytosine
Thymidine	α*-*glutamylthymidine
	α-putrescinylthymine
Uracil and modifications	ß-glucopyranosyloxymethyluracil (base J)
	uracil
	5-dihydroxypentauracil
	5-hydroxymethyldeoxyuracil
Others	deoxyarchaeosine
	2,6-diaminopurine

Sulfamethazine, an inhibitor of folate synthesis, can suppress gene-silencing in plants (Zhang et al., 2012). In the same genetic screens for suppressors that would release the silenced expression of the double reporters (*LUC* and *NPTII*) in the mutant *ros1* (*Repressor of Silencing 1*), extragenic suppressor mutations mapping to *FOLYLPOLYGLUTAMATE SYNTHETASE 1* (*FPGS1*) were identified (Zhou et al., 2013). *In vivo*, folate can be polyglutamylated and FPGS catalyzes the sequential conjugation of additional *r*-linked Glu residues to the initial Glu. The *fpgs* mutation reduced the total folate abundance and DNA methylation in all three cytosine contexts, as well as the level of the repressive mark H3K9me2. Similar to the example of vitamin C in animal cells, here, adding to the plant medium a stable form of folate (5-formyltetrahydrofolate, 5-CHO-THF) could revert the short-root phenotype and the recovery of kanamycin resistance from the reporter *NPTII*. This treatment also restored the level of H3K9me2. Moreover, the addition of 5-CHO-THF to the wild-type plants also increased the DNA methylation level, but that of H3K9me2 remained constant. These results suggest that the level of DNA methylation during normal development of the plant is directly limited by folate, with a secondary consequence on histone methylation. Over-expressing methionine synthase, to increase the precursor to SAM, represses resistance to *Pseudomonas syringae* DC3000 and genome-wide increase in DNA methylation, reinforcing the importance of folate one-carbon metabolism for correct DNA methylation and its dynamics in responding to stress stimuli (Gonzales and Vera, 2019).

The locus *SUPPRESSOR OF DRM2 CMT3* (*SDC*) is silenced conjointly by the DRM2- and CMT3-mediated methylation pathways. Thus, while *SDC* is expressed in the double mutant *drm2 cmt3*, it is silenced in the wild-type and also in each of the two respective single mutants. Using the transgene *SDCpro-GFP* as the reporter, which was silenced in the WT and *cmt3* background, one mutant was identified with alleviated reporter gene suppression in the WT background. The mutation was mapped to the gene (*MTHFD1*) encoding METHYLENETETRAHYDROFOLATE DEHYDROGENASE/METHENYLTETRAHYDROFOLATE CYCLOHYDROLASE 1 involved in the interconversion of three key folate intermediates, and other functions including the production of purine, pyrimidine, and SAM (Groth et al., 2016). This mutation led to the genome-wide loss of CHG and CHH methylation, reduced H3K9me and re-activation of mobile elements. The actual cause of these consequences, however, may not be the mere results of diminished folate pools, because, in contrast to *fpgs1* above, applied 5-formyltetrahydrofalte (5-CHO-THF) led to rather root growth inhibition of the *mthfd1-1* (but not on WT seedlings). Treatment with 5-CHO-THF as well as 5-CH_3_-THF did not rescue the DNA methylation defects in *mthfd1-1*. The authors have attributed the cause to be inhibition of methionine synthase.

In human cells, a fraction of the MTHFD1 is recruited to chromatin by a direct interaction with the histone acetyl reader Bromodomain-containing protein 4 (BRD4), whose deregulation has been linked to oncogenesis (Sdelci et al., 2019). Thus, besides SAM production, the MTHFD1 protein itself, involved in nuclear folate metabolism, can be a component of chromatin complexes that directly influence gene expression.

### Epi-Transcriptomics-m^6^A, With Links to TCA Cycle, Methylated DNA and Acetylated Histones

RNA containing modified bases had been noted since the 1970s (Desrosiers et al., 1974). Because mapping modified RNA bases was labor-intensive (https://bitesizebio.com/13550/a-short-history-of-sequencing-part-1-from-the-first-proteins-to-the-human-genome/), it took a backseat to the easier task of determining the actual sequences themselves, aided by the technical advances in DNA sequencing. In the process of copying the RNA into cDNA by supplying the standard nucleotides in the *in vitro* reactions, any chance of following up on modified RNA bases quickly dropped out of the radar, which lasted over 3 decades.

Over 150 different chemical modifications are known, most of them are in structural RNAs (e.g. tRNA, rRNA), but a few are also present in mRNA (http://modomics.genesilico.pl/ and http://mods.rna.albany.edu). The prevailing idea is that these modifications determine the post-transcriptional fate of the RNA (Boccaletto et al., 2018; Hu et al., 2019). But it is likely that at least some of the modifications on RNA, particularly methylation, can compete with the demands by DNA and histones. Only m^6^A will be discussed here.

The m^6^A found in DNA is also a major mark on total RNA (0.1% to 0.2% of the nucleotide). It maps preferentially to the transcription start sites, the stop codon, and the 3’ UTR. Compared to its counterpart on DNA, for which the functions are speculative with the possible exception of double-strand breaks, we have better ideas about its effects on RNA. The first-ever hint of its functional importance at the whole-organism level was distilled from disrupting the gene *MTA* (At4g10760), encoding the mRNA adenosine methyltransferase, in Arabidopsis (Zhong et al., 2008). Homozygous T-DNA insertion mutant embryos are white and fail to mature beyond the globular stage, indicating that the optimal m^6^A level must be connected with a number of elementary metabolic networks.

The levels of m^6^A in RNA are positively correlated with transcript stability. This may be one mechanism to enhance the half-life of selective mRNAs. This hypothesis is clearly supported by a mammalian model system, in which viral infection stimulated the binding of a specific RNA m^6^A demethylase, ALKBH5, to an important transcript–the α-ketoglutarate dehydrogenase (*OGHD*) mRNA. This binding is correlated with blocking viral infection. ALKBH5 is a methylated protein, and demethylation at its R107 inactivates its catalytic activity (Liu et al., 2019). In this alternative scenario, the *OGHD* mRNA became hypermethylated as well as stabilized as the result. The higher OGHD also stimulated the production of the metabolite, itaconate, an intermediate of the TCA cycle, required for viral replication. RNA m^6^A demethylases are actually α-ketoglutarate-dependent dioxygenases, indicating their catalytic activities are linked to those of the TCA. They can erase not only methyl but also alkyl groups on their diverse types of targets. For example, in animals, ALKBH1 can act on RNA, DNAs, and histones. This dioxygenase also targets several m^1^A methylated tRNAs in the mitochondria, influencing organellar translation (Kawarada et al., 2017; Muller et al., 2018).

In Arabidopsis, there are 13 different ALKBHs. Most of them are localized in the nucleus and in the cytoplasm, except ALKBH1D, which is chloroplastic (Mielecki et al., 2012). ALKBH10B is the principal mRNA m^6^A eraser influencing floral transition by controlling the transcript levels of *SPL3*, *SPL9* and *FLOWERING LOCUS T* (Duan et al., 2017). ALKBH9B also seems to play roles in plant defense against pathogens (Martinez-Perez et al., 2017). In rice, *ALKBH1*, *ALKBH6*, *ALKBH8B*, and *ALKBH10A* were found to be differentially regulated by drought, cold or ABA. All of these observations are consistent with the notion that these ∝-ketoglutarate-dependent demethylases may have roles as environmental sensors (Hu et al., 2019). Thus, there seems to be a possible coordinated switch of the chromatin towards more of an active state by demethylation of DNA, RNA, histones, coupled to acetylation of histones.

### Metabolite Methylation: Functions and Influence on Chromatin State

In mammals, methylation of phosphotidylethanolamine (a phospholipid) was experimentally shown to divert methylation from histones and a protein phosphatase2A. This overall process is thought to reflect optimization of sulfur metabolism as well as transcriptional regulation of sulfur and the expression of genes involved in phospholipid metabolism (Ye et al., 2017). These biochemical results prove that metabolites are parts of the global methylome by competing for SAM away from histones and non-histone proteins.

Plants are metabolite factories (Fang et al., 2019), yet their potential connection with epigenetics has hardly been explored. Examples of well-known metabolites are methyl-salicylic acid and methyl-jasmonate, with broad physiological effects on development and plant–environment interactions (Vogt, 2010; Hu et al., 2017). *O*-methyltransferases have been implicated in lignin biosynthesis (Zhong et al., 1998) and in modifying flavonoids as well as esters with aromatic vicinal dihydroxyl groups (Ibdah et al., 2003). Methylation of certain anthocyanins alters the intensity of pigment hues, generating their diversity in stability and functions, likely related to their function as protectants against UV, pigmentation, antioxidation or attractants of insects (Du et al., 2015). In Arabidopsis and the cyanobacterium *Synechococcus*, trimethylation of glycine yields glycinebetaine, which confers tolerance to experimental stresses that include low temperature, drought and high salinity (Waditee et al., 2005). The enhanced salt tolerance in the case of *Synechococcus* can be dramatic, as illustrated by the transgenic expression of two glycine methyltransferases that enabled the cells to grow in 0.6 M NaCl, converting a fresh-water organism into a marine species (Waditee et al., 2005). Methylglycine also exists in mammalian cells, which converts the amino acid into an oncometabolite. *N*-methylglycine or sarcosine stimulates the invasion and aggressiveness of prostate cancer cells (Sreekumar et al., 2009). *1*-Methylnicotinamide is known to be a developmental regulator of prostacyclin synthesis in mammals (Chlopicki et al., 2007). More recently, this metabolite is also found to be required to maintain stem cells in their embryonic state by competing with the deposition of H3K27me3 marks (Sperber et al., 2015). Even the SAM-independent methyl donor, folate, itself, can be methylated, which is the active form of vitamin B9 used by the human body in circulation. As it is generally thought that the number of metabolites vastly outnumbers that of proteins (Li and Snyder, 2011), metabolites could turn out to be extremely tenacious competitors for SAM, and thereby, also chromatin structures.

Arsenate induces the accumulation of several methylated/citrullinated proteins [FUS, EWS, and TAF15; see (Tanikawa et al., 2018)]; arsenate is itself inactivated by methylation (Tseng, 2009). Methylhalides (organic halogens) are produced by a large number of ecosystems, crops, and biota. Likewise, the terrestrial source of methyl iodide can account for 80-110x10^9^ tons of iodine per year (https://www.sciencedirect.com/topics/chemistry/methyl-halides) (Carpenter, 2015). Despite little is known about their biological roles, there are nonetheless methyltransferases that have already been identified in plants, such as *Endocladia muricata* (marine red alga), *Phellinus promaceus* (white rot fungus) or *Mesembryanthemum crystallanium* (ice plant), that can use SAM to methylate organic ions such as the anions iodide, chloride, bromide (Wuosmaa and Hager, 1990) and the amino acid alanine (Barkla and Vera-Estrella, 2015), possibly associated with various adaptive functions. Along with the thinking of diets in humans being linked to epigenetics and diseases, one wonders whether plants metabolites, liberated after being ingested by the organism, can directly be used by the animal epi-genome as well. This hypothetical scenario is plausible; it would be analogous to horizontal gene transfer (bacteria taking up free DNA) or interspecific exchanges of small regulatory RNAs between pathogens and plants [for an example, see (Hudzik et al., 2020)].

### Heterochromatin Protein1 and Histones-Collaborators or Competitors?

The methyl marks on histones are “read” by protein modules, which then translate them into specific gene expression profiles. One of the better-characterized methyl-lysine readers in plants is LIKE HETEROCHROMATIN PROTEIN1 (LHP1), a Chromo domain protein in the Polycomb Repressive Complex 1 (PRC1). The mutant *lhp1* displays pleiotropic phenotypes that include abnormal rosette leaves, smaller plant stature, partial sterility, early flowering and a terminal flower (Gaudin et al., 2001; Kotake et al., 2003). The mutation has an impact on general metabolism: in a genetic screen for mutants affected in the synthesis of glucosinolates, *tub8* was identified. Intriguingly, this mutation turned out to be allelic to *lhp1* (Kim et al., 2004).

While a “reader” has an important role in translating a histone mark into a cellular function, histones and (L)HP1 may also have complicated relationships in that they compete for the same metabolites for adducts. LHP1 binds to trimethylated H3K27, which is a euchromatin mark *in vivo* (Turck et al., 2007; Zhang et al., 2007) and sensitive to the cytosolic level of acetyl-CoA. The name LHP1 had been inspired by its notable sequence similarity to the founder HETEROCHROMATIN PROTEIN1 (HP1) of Drosophila. HP1 was identified as *Su(var)205*, a suppressor mutation of Position Effect Variegation (PEV) (Tschiersch et al., 1994; Lloyd et al., 1999; Elgin and Reuter, 2013). For clarity, PEV describes the variegation of a phenotype caused by a wild-type gene’s abnormal juxtaposition to heterochromatin brought about by either chromosomal rearrangement or transposition. One model suggests that heterochromatin can spread from HP1-bound sites to adjacent regions with stochastic endpoints, suppressing the expression of neighboring genes in some cells but not in others. Modifiers of PEV almost always corresponded to deleted or disrupted loci encoding histone, chromatin proteins and components in RNA interference. One surprising result, at least at the time, from these genetic screens was the discovery of *SUPPRESSOR OF ZESTE5* [*Su(z)5*]. Instead of a mutation in yet another locus encoding a chromatin-related protein, *Su(z)5* turned out to impair the synthesis of SAM (Larsson et al., 1996). With hindsight, it is obvious that SAM is the limiting methyl donor for a wide range of molecules, including chromatin proteins and metabolites. But in the absence of this knowledge at the time, PEV suppression was hypothesized by the authors to be linked to the reduced levels of certain polyamines, which was empirically confirmed.

*In vivo*, the Arabidopsis LHP1 is found in nuclear speckles (Gaudin et al., 2001; Kotake et al., 2003), which represent an example of non-membrane bodies. However, whether these speckles reflect the intrinsic ability of LHP1 to self-aggregate and to undergo liquid–liquid phase separation, or coerced into foci by association with other cellular components, is not known. We raised this possibility because the Drosophila and one of the human homologs have an intrinsic ability to phase separate *in vitro* (Larson et al., 2017; Strom et al., 2017). In the latter case, the *in vitro* phase separation HP1 isoform α was correlated with phosphorylation on the protein’s N-terminal extension, or alternatively, by the presence of certain ligands including non-specific DNA and spermine (Larson et al., 2017). This phase separation into protein droplets has been interpreted by the authors as recapitulating the key role of HP1 as nucleation sites for chromatin condensation and spreading that occur *in vivo*. HP1α and the Drosophila homolog are endowed with an ability to self-assemble into a large oligomer, and nucleating around charged molecules. However, the *in vivo* physiochemical conditions facilitating these particular HP1s to assume gel-like droplets are still a matter of debate [see (Richter et al., 2008) for an example of conditions]. Because spermine and other polyamines are abundant metabolites in cells and can condense nucleic acids, these polycations may ease HP1 into molecular crowding and local phase separation.

**Figure 5:**
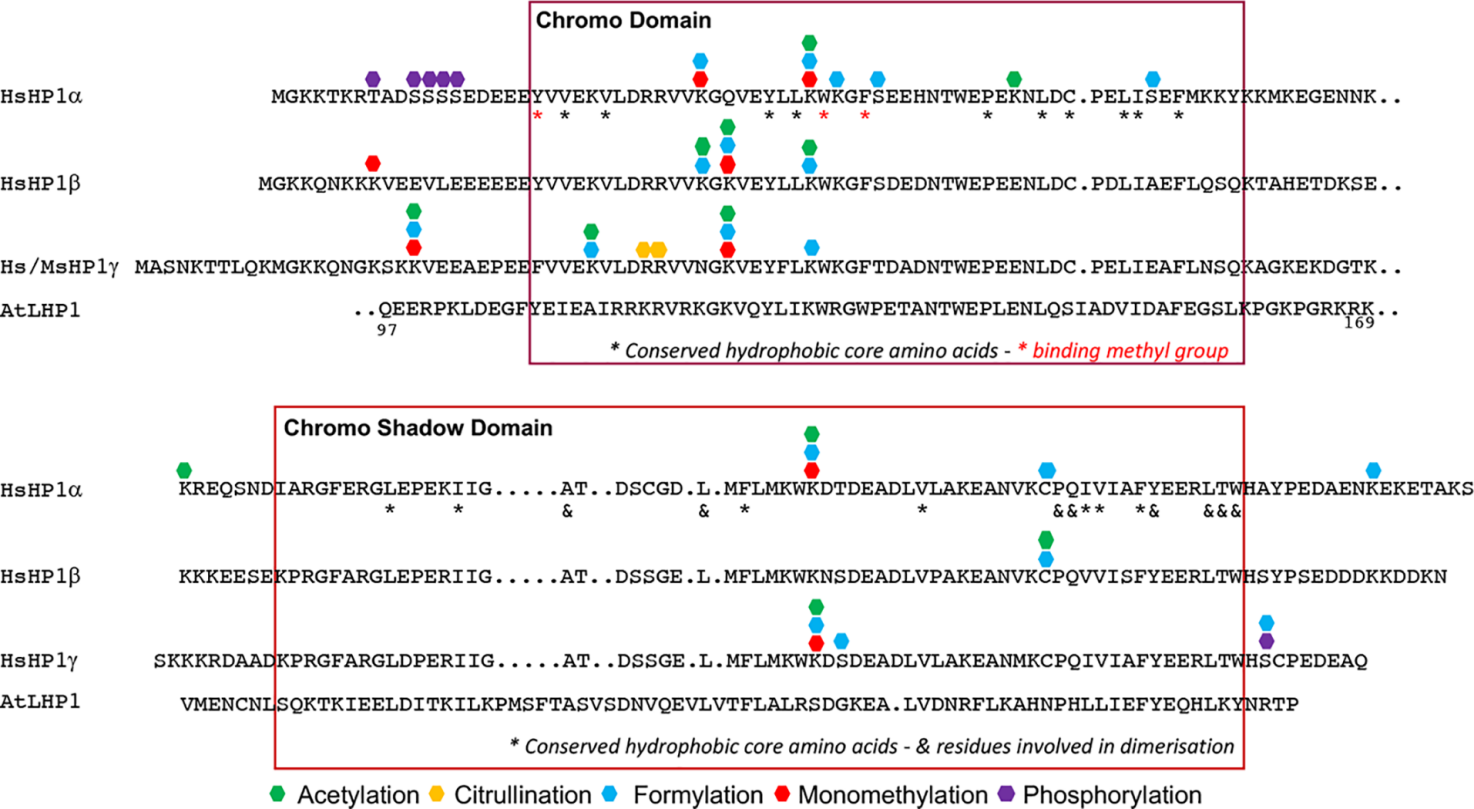
Modified residues in Chromo and Chromo Shadow domains of HP1 family. The figure was adapted from Gaudin et al. (2001); LeRoy et al. (2009); Wiese et al. (2019). Conserved residues involved in methyl lysine binding and hydrophobic cores were deduced from Nielsen et al. (2002); Fischle et al. (2003).

There are covalent modifications on histones–acetylation, methylation, citrullination, and formylation–that are also predicted on all three human HP1 isoforms (LeRoy et al., 2009; Wiese et al., 2019). Most of the target lysines are conserved in the Arabidopsis LHP1 (**Figure 5**). The role of histone formylation in influencing gene expression is not completely understood despite that protein formylation in eukaryotes is widespread (Wisniewski et al., 2008). Nonetheless, formylation of histones (**Figure 1**), and chromatin proteins such as High-Mobility Group (HMG) and HP1 homologs, adds an additional point of convergence between epigenetics and metabolism. Deformylases (Meinnel et al., 1996) and formyl-binding chemosensory receptors (Dahlgren et al., 2016) exist in animals, suggesting dedicated metabolic and signaling processes, likely in responding to stressful stimuli. The metazoan HP1s are also recruited to damaged DNA; thus, it has been proposed that formyl donors could come from 3’-formylphosphate, a highly reactive intermediate generated from oxidation of the 5’-deoxyribose in the damaged DNA (Jiang T. et al., 2007; Ayoub et al., 2008; LeRoy et al., 2009; Luijsterburg et al., 2009). Are these processes also inherent to normal development? For example, would (L)HP1 be involved in the repair of hundreds of double-strand breaks generated regularly during meiosis? We have also mentioned that LSD histone demethylases are huddled around sites of DNA damages (section *Histone Methylation and Metabolic Cofactors*), whether there is functional cooperation with (L)HP1 in DNA repair is not known.

## Summary and Prospects

In his book, “Who Rules the World?”, the acclaimed MIT linguist, Noam Chomsky, weighs in on the question of how the term of the global discourse is set. The “Who” is somewhat of an abstraction, but generally personified as a “Deep State”, a kind of sprawling network that knows how to control the most “vital strategic points”.

**Figure 6:**
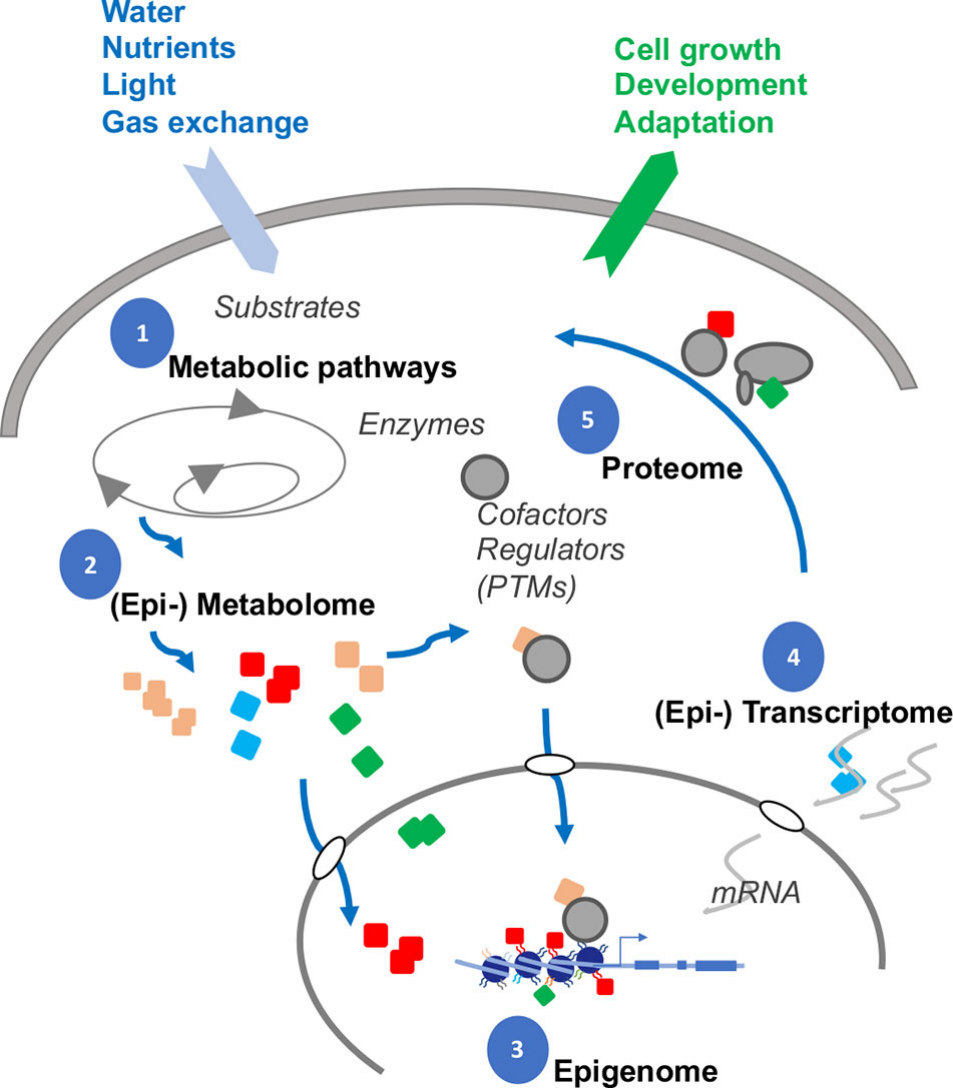
Interplay between DNA, RNA, chromatin and metabolites. Gene expression is dynamically modulated by feedback with interdependent subcellular systems (numbered). Many metabolites (colored symbols) are substrates for chemical attachments. The cellular compositions of the metabolites are likely altered, as adaptation, to nutrient and water availability, light perception, gas exchange, and interactions with the rhizosphere. In turn, the metabolites themselves can attach, either covalently (adducts) or by electrostatic interactions (cofactors), to proteins, RNAs, and chromatin to shape the final gene expression profiles in responding to changing developmental and environmental stimuli (output arrow).

One vital strategy evolved in cells to coordinate complex workings is epigenetics, the powerful driving force behind multicellularity, pluripotency and the capacity to adapt to new environmental niches (**Figure 6**). In just the last few years, torrents of never before seen modifications have been revealed in the genomes of animals. Most of these newer adducts in **Figure 1** are beyond the remit of this review, but it does reveal the sprawling network of possible interactions between epigenetics and metabolites. Neither can **Figure 1**–already detail-rich– be exhaustive, as new modifications are being continually discovered.

The newer sequencing technologies are also identifying novel modifications on RNA and DNA. Cytosines modified by formylation, carboxylation or hydroxylation have now been found in the human genome. These discoveries also immediately raise questions on how these marks influence the local DNA structure? What are the instructions encoded by such modified DNA? Do they mark special sites on the DNA, such as breakpoints? Do these DNA modifications influence the local deposition of histone marks? If so, are there particular histone marks that might be more susceptible?

We have dealt mainly with the donors, acetyl-CoA, and SAM, but it is clear that their implications are deep. Acetyl-CoA is the starting point of fatty acid synthesis and membranous structures from carbohydrates. Acetyl-CoA in the cytosol is fed into the synthesis of a plethora of small chemicals, many of which have proven to be important for plant growth, development and responding to environmental cues. Methylation also regulates many cellular processes. We are familiar with methyl-jasmonate and methyl-salicylic acid. But in fact, myriads of small molecules can exist in methylated variants, suggesting that all of them, could have an impact on altering epigenetic regulation by competing for SAM. How the novel metabolites are replenished, by which pathways, is of obvious interest.

How specific gene expression patterns emerged from the complex interactions between chromatin and the immense arrays of metabolites (**Figure 6**) will be a daunting, but an extremely timely, question concerning food security, as we attempt to rationally design crops that can adapt better to drought and high temperatures. We are still largely in the awakening phase of discovering and cataloging the types of modifications. Identifying and characterizing the roster of proteins that interact with specific metabolites will be equally a critical step towards understanding how the metabolites may influence epigenetics. Because metabolites are of such diverse biochemical and physical nature, functional analyses will require dedicated techniques of sufficient spatial resolution, sensitivity, and discrimination that will permit tracking a single class of metabolites in a single cell before we can correlate them to the nuclear output.

## Author Contributions

JL and VG conceived the review. JL wrote the manuscript. VG revised the manuscript. JL and VG designed the figures.

## Conflict of Interest

The authors declare that the research was conducted in the absence of any commercial or financial relationships that could be construed as a potential conflict of interest
